# T cell responses to control fungal infection in an immunological memory lens

**DOI:** 10.3389/fimmu.2022.905867

**Published:** 2022-09-13

**Authors:** Jaishree Sharma, Srinivasu Mudalagiriyappa, Som Gowda Nanjappa

**Affiliations:** Department of Pathobiology, College of Veterinary Medicine, University of Illinois at Urbana-Champaign, Urbana, IL, United States

**Keywords:** CD4+ T cells, CD8+ T cells, memory, fungal, vaccination, infection, immunity

## Abstract

In recent years, fungal vaccine research emanated significant findings in the field of antifungal T-cell immunity. The generation of effector T cells is essential to combat many mucosal and systemic fungal infections. The development of antifungal memory T cells is integral for controlling or preventing fungal infections, and understanding the factors, regulators, and modifiers that dictate the generation of such T cells is necessary. Despite the deficiency in the clear understanding of antifungal memory T-cell longevity and attributes, in this review, we will compile some of the existing literature on antifungal T-cell immunity in the context of memory T-cell development against fungal infections.

## Introduction

An increasing global burden of fungal diseases due to increasing immunocompromised individuals has heightened the need for effective preventive and therapeutic strategies. Fungi are one of the large biome classes, but only a handful of them are pathogenic to humans, causing a significant case fatality of up to 90%. More than 150 million severe cases and over 1.5 million succumb to fungal infections annually, despite the use of antifungal drugs (1–3). Some existing antifungals are effective but cause serious side effects and are liable to the growing drug-resistant fungal pathogens. With expanding knowledge on host–fungal pathogen interactions, there is a tremendous leap in the thrust to develop fungal vaccines. The pan-fungal vaccine is highly desirable, but the features of different fungal pathogenesis and elicitation of distinct immune responses require a clear understanding of the fungus–immune system interface, i.e., vaccine immunity and the potential to develop immunological memory. This review gives an overview of antifungal memory T cells.

Although there is a good amount of evidence of antibody-mediated immunity (4), adaptive immune cell responses against pathogenic fungi are mainly mediated by T cells, and genetic or acquired T-cell deficiency leads to a higher incidence of opportunistic infections (1, 5, 6). Antifungal defense mechanisms by CD4^+^ T cells, a major class of T cells, involve the secretion of proinflammatory cytokines and cell–cell interactions to activate innate immune cells, help CD8^+^ T cells, and provide help for the generation of antibodies from B cells (7). The antifungal CD4^+^ T-cell immunity involves the expression of IFNγ, TNFα, GM-CSF, and IL-17A cytokines, which are differentially produced in a fungus- and tissue-specific manner. For example, IFNγ, TNFα, and GM-CSF are predominantly induced during histoplasmosis, aspergillosis, cryptococcosis, paracoccidioidomycosis, pneumocystosis, and talaromycosis, whereas type 17 cytokines, IL-17A/F, and IL-22 are mainly induced during candidiasis, coccidioidomycosis, blastomycosis, and mucormycosis (reviewed here). Nonetheless, it is common to see both types of responses with variable degrees in most fungal infections. These secreted cytokines generate an inflammatory milieu and act on other cells for innate cell recruitment, activation, secretion of antimicrobial peptides, and killing of fungi (8–10). In contrast, the antifungal T cell-mediated immunity is compromised if their cytokine signature yields regulatory or unprotective cytokines that can lead to severe disseminated infections (11, 12). Despite the need for CD4^+^ T-cell help for CD8^+^ T-cell activation and memory maintenance in viral and bacterial infection scenarios, using mouse models of fungal infections against *Pneumocystis*, *Histoplasma*, and *Blastomyces*, the studies have shown that antifungal CD8^+^ T cells can be induced, retained as long-lasting memory, and recalled upon the challenge to provide immunity independent of the T-cell help during mouse models of *Pneumocystis*, *Histoplasma*, and *Blastomyces* infections (8, 13–16). Antifungal activity of CD8^+^ T cells involves cell cytotoxicity (17) and secretion of proinflammatory cytokines; the latter often mimics CD4^+^ T-cell antifungal cytokine functions.

The host’s first response to fungal invasion starts with innate immunity, which then engages the adaptive immune arm to mount antigen-specific responses to control or clear fungal infection (18). The pattern recognition receptors (PRRs) are critical for innate immune responses for initial fungal control, and their mutations are associated with higher susceptibility (6, 19–21). The activation of innate immune cells and generation of apt inflammatory milieu facilitate dendritic cell priming of naïve T cells to become effectors, which eventually differentiate to form antifungal memory T cells (22). Thus, the innate immunity dictated by fungal recognition shapes adaptive T-cell immunity and immunological memory.

## Fungal recognition by the immune system: Bridging innate to adaptive immunity

Among innate cells, dendritic cells are essential for priming naïve T cells (23). The activated dendritic cells process and present the antigens to CD4^+^ and CD8^+^ T cells through MHC-II and MHC-I molecules, respectively. Along with antigen presentation, dendritic cells provide costimulatory signals for T-cell responses (24). Thus, the functions of dendritic cell maturation and activation are a critical step toward bridging innate with adaptive immunity, and such events are mainly mediated by PRR signals. PRRs are a category of host cell receptors that sense specific molecules/patterns, the pathogen-associated molecular patterns (PAMPs) such as β-glucans and mannans of pathogenic fungi, and this recognition is key for innate immune cell activation to provide a primary antifungal defense. The PRRs are mainly classified as Toll-like receptors (TLRs), C-type lectin receptors (CLRs), retinoic acid-inducible gene I-like receptors (RLRs), and NOD-like receptors (NLRs), which can directly bind to the PAMPs of fungi, whereas damage-associated molecular patterns (DAMPs) can bind to PRRs and their canonical DAMP-sensing receptors such as P2X purinoceptor 7 (P2XR7), triggering receptor expressed on myeloid cells 1/2 (TREM1/2), and receptor for advanced glycation end products (RAGE) (25–28). Several of PAMPs of fungi, including β-glucans, mannans, glycoprotein A, and glyceroglycolipids, have been identified for their functions using their PRRs in the host (29–32). There are excellent reviews on PRRs and fungal immunity elsewhere. Here, we highlight how PRRs can influence the innate immune cells to guide adaptive T-cell immunity.

Although negative signaling is noted with few PRRs, many are associated with their positive signaling to promote activation, phagocytosis, and antigen presentation by dendritic cells to T cells. The activated innate immune cells generate an inflammatory micro milieu conducive to the recruitment, activation, differentiation, and expansion of fungal-specific T cells by secreting cytokines and chemokines. Among PRRs, fungal-recognizing CLRs are instrumental in driving innate immune cell responses. Due to structural differences, different fungi show differential CLR binding properties leading to diverse host cell responses. The prototypic member of this family, the Dectin-1 receptor, expressed on innate immune cells including macrophages, neutrophils, and dendritic cells (DCs), recognizes β1-3-glucans of the fungal cell wall. The interference of Dectin-1 interaction with β-glucans by a soluble dectin-Fc fusion protein dampened the expression of inflammatory cytokines, TNFα, IL-1, IL-6, MIP-2, CCL3, G-CSF, and GM-CSF, expression *in vivo*, and increased fungal burden during aspergillosis (33). Ablation of Dectin-1 resulted in decreased reactive oxygen species (ROS) production by neutrophils and the ability to kill *Aspergillus in vitro.* Additionally, alveolar macrophages of Dectin-1^−/−^ mice had defective production of proinflammatory cytokines and chemokines, including IL-1α, IL-1β, and TNFα. The Dectin-1 recognition of *Aspergillus* seems important for IL-17A production, and neutralization of IL-17A led to impaired *Aspergillus fumigatus* clearance and higher mortality of infected mice (34). Dectin-1 promoted the survival of antigen-specific CD4^+^ T cells, not the CD8^+^ T cells, specifically in GI-associated lymphoid tissues following systemic *Candida* infection, and ablation of Dectin-1 reduced the tissue-specific dendritic cells and increased activation of CD4^+^ T cells leading to higher susceptibility to *Candida*-induced colitis (35). During systemic *Candida* infection, the protective role of Dectin-1 was fungal strain-specific, possibly due to variable adaptation of *Candida albicans* strains *in vivo*, including the changes in the microbiota of mice due to different mouse facilities, with changes in the cell wall components and high chitin in the cell wall masks the dependability on Dectin-1 recognition (36). Further, pathogenic fungi can avoid host Dectin-1 recognition of β-(1,3)-glucan by masking with α-(1,3)-glucan, phosphatidylserine, capsule, rodlet layer/melanin, and mannans or trimming to reduce the exposure in the cell wall, thus increasing immune evasion *in vivo* (37–40). However, Dectin-1 was dispensable for controlling infections from *Blastomyces*, *Cryptococcus*, certain strains or species of *Candida*, or *Candida* colonization (36, 41–45), suggesting the differential requirement of CLRs for fungal immunity.

Unlike the Dectin-1 receptor, cytoplasmic domains of Dectin-2 and Mincle receptors lack their own ITAM motifs and associate with FcRγ immunoreceptor harboring cytoplasmic ITAM motif for signaling (46, 47). Dectin-2 and Mincle have been shown to be important for immunity against blastomycosis (48), aspergillosis (49), histoplasmosis (44), chromoblastomycosis (50), disseminated candidiasis (51, 52), and species-specific candidiasis (45). Dectin-2 signals through the Syk-CARD9 pathway and promote Th17 cell responses (51) by inducing the expression of IL-1 and IL-23 cytokines (53). Despite that Dectin-2 and Mincle share their signaling through FcRγ immunoreceptors, their role in activation, expansion, and differentiation of antigen-specific CD4^+^ T-cell responses may differ. While Dectin-2 was essential for enhancing Th17 cell differentiation, Mincle recognition suppressed Th17 polarization during chromoblastomycosis (50).

In addition to CLRs, TLRs expressed by innate cells are involved in the control of fungal infection. Myeloid differentiation primary response protein 88 (MyD88), an adaptor molecule for many TLRs signaling, has been shown to play a role in antifungal immunity against *Blastomyces dermatitidis*, *Paracoccidioides brasiliensis*, *A. fumigatus*, *Cryptococcus neoformans*, and *C. albicans* (54–56). TLR2 plays a significant role in conferring protective immunity against *Candida* infection at mucosal sites, including gastrointestinal and reproductive tracts by inducing Th17 differentiation through MyD88 signaling (57, 58). However, the role of TLR2 in controlling systemic candidiasis seems to be fungal strain specific (59, 60). Additionally, IL-1R/MyD88 signaling pathway is necessary for host resistance against *Candida*, and TLR4/MyD88 pathways mediate protection against *Aspergillus* infection by regulating Th1 and Th2 response (54, 61). TLR3 in DCs senses fungal RNA derived from dying cells and potentiates the cross-presentation to activate CD8^+^ T cells during aspergillosis (62). Nevertheless, compared to those of CLRs, the functions of many TLRs in the context of antifungal immunity seem to be modest or redundant.

NLRs function against fungal defense mainly involved the activation of inflammasomes, which leads to caspase-dependent production of functional IL-1β and IL-18 cytokines. Both of these cytokines have been shown to exert antifungal host defense in an NLRP3-dependent manner (63). NLRC4 negatively regulates NLRP3 inflammasome activity, suppressing early IL-1β and late IL-18-mediated antifungal CD8^+^ T-cell responses during pneumocystosis (64). Thus, some PRRs of the non-CLR class play a role in immunity against fungal infections (26).

Genetic polymorphisms are associated with susceptibility or resistance to infections. The genetic TLR polymorphisms related to fungal disease susceptibility in humans undergoing allogenic stem cell transplants seem to be modest or minimal (65, 66). The genetic predisposition due to PRR polymorphisms to fungal infections is variable and depends on the pathogen or the degree of inflammation. For example, TLR4 polymorphism D299G is associated with increased susceptibility to *Candida* bloodstream infection, possibly due to higher immunosuppressive cytokine IL-10 production. However, such susceptibility was not seen in urogenital *Candida* infection (67). Similarly, the TLR4 polymorphisms (D299G/T399I), despite the normal colonization of the fungus, are associated with mitigating the hyperinflammation and tissue damage during aspergillosis (66). Similarly, genetic polymorphisms of CLRs have been associated with susceptibility to fungal infections. Dectin-1 single-nucleotide polymorphisms (SNPs), rs3901533 and rs7309123, enhanced the susceptibility to invasive pulmonary aspergillosis (68). Dectin-1 polymorphism of Y238X led to decreased receptor signaling and increased susceptibility to invasive aspergillosis and recurrent vulvovaginitis caused by *Candida* (69, 70). Alternative splicing leading to truncated Dectin-1 seen in the C57BL/6 strain, compared to the DBA/2 mouse strain, increased the susceptibility to coccidioidomycosis (71). Thus, it is essential to decipher gene polymorphisms in humans to understand the susceptibility to fungal infections.

T cells can also express several PRRs to respond to PAMPs during fungal infections. Engagement of the cell-intrinsic PRR pathway is one of the non-classical T-cell signaling routes to enhance the activation, effector function, and memory formation of T cells as proposed originally by Janeway (72). Important PRRs on T cells that detect fungal PAMPs are TLRs, NLRs, and damage-associated molecular pattern-sensing receptors. TLRs can function as co-stimulatory receptors that complement TCR-induced signals to enhance effector T-cell proliferation, survival, and cytokine production (73). T cells expressing TLRs, including TLR2 and TLR4, can directly sense pathogens and modulate T-cell responses. Naïve CD4^+^ T cells do not express significant levels of TLR2/TLR4 mRNA and proteins, but activated and memory T cells express high levels of membrane-bound TLR2 and TLR4 (74, 75). TLR2 signaling in T cells can be modulated by TCR and IL-2-induced mTOR signals (76). Intrinsic MyD88 signaling can modulate the T-cell functions during fungal infections. MyD88 signaling, both extrinsic, non-CD4^+^ T cell-mediated (77) and intrinsic, CD8^+^ T cell-mediated (78), fosters fungal vaccine immunity by T cells by regulating the survival and proliferation of effector T cells. MyD88 promoted the sustained Tc17 cell proliferation by activating mTOR *via* Akt1, and cell-intrinsic IL-1R and TLR2 signaling, but not IL-18R, were required for MyD88-dependent Tc17 responses (78). MyD88 deletion in FoxP3^+^ regulatory T cells increased the fungal burden and immunopathology during oral *C. albicans* infection in mice, coinciding with reduced IL-17A expressing FoxP3^+^ T cells (T_reg_17) and increased dysfunctional IFNγ^+^/FoxP3^+^ cells (IFNγ^+^ T_reg_). This dysregulated IL-1β-mTOR-Treg17 axis contributes to overt inflammation during mucosal infections in elderly individuals in a model of oral candidiasis (79). NLRP3, a member of the NLR family, can indirectly sense danger signals. In a murine model of disseminated talaromycosis, compared to wild-type mice, Casp-1 and Nlrp3 global KO mice displayed higher mortality rates and fungal load, which correlated with impaired CD4^+^ T-cell recruitment into granulomas (80). Although this study did not look into T-cell intrinsic effect, NLRP3 signaling in CD4^+^ T cells has been shown to augment Th1 immunity (81). Further studies are needed to dissect the T-cell intrinsic PRR functions against fungal infections. Interestingly, PRR has been used to generate modified TCR of T cells. Dectin-1-chimeric antigen receptor (D-CAR) was bioengineered using the extracellular domain of Dectin-1 to redirect T-cell specificity toward fungal β-glucan moieties for immunity (82). D-CAR+ T cells could inhibit *A. fumigatus* hyphae formation *in vitro* and reduce pulmonary fungal burden *in vivo.* In this study, chimeric CD8^+^ T cells kill the fungi directly by pumping out cytolysins onto yeast/hyphae of *Aspergillus* and indirectly by secreting IFNγ that can potentiate the killing of yeasts by neutrophils (83).

## Antifungal T cells

CD4^+^ T cells, also called helper T cells, are instrumental in controlling fungal infections, and their deficiency leads to severe disseminated infections by opportunistic fungal pathogens. As the name suggests, the helper cells bolster innate immune cell functions, aid in the generation of productive B-cell responses, help CD8^+^ T-cell responses, and control autoimmunity. Based on their cytokine secretion and functions during fungal infections (84, 85), T cells are classified into Th1 (IFNγ, GM-CSF, and TNFα), Th17 (IL-17A/F), Th22 (IL-22), Th2 (IL-4 and IL-13), Th9 (IL-9 and IL-10), Treg (IL-10 and TGFβ), and Tr1 (IL-10). The cytokines produced by T cells have a multifaceted role in controlling or regulating the pathogenesis during infections, including fungal infections (86). Although a mixture of different cytokine-producing T cells is often found during fungal infections, the predominant subset of T-cell responses is associated with the type of pathogen, infection, or tissue location (**Table 1**). Here, we will highlight some of the roles of these T cells for immunity against different pathogenic fungi with a focus on memory phenotypic cells.

**Table 1:** Major T-cell subsets elicited and shown to be protective against fungal pathogens.

Pathogenic fungi	Mechanism(s) of protection			References
***Aspergillus* spp.**	Th1 (circulation), Th17 (lungs)	Tc1		(87–89)
***Blastomyces* spp.**	Th1, Th17	Tc1, Tc17	Tc1 (_EM/CM_) Tc17 (_EM_)	(56, 90)
***Candida* spp.**	Th1, Th17	Tc1, Tc17	T_RM_	(91–94)
***Cryptococcus* spp.**	Th1, Th17?	Tc1	T_RM_	(95, 96)
***Coccidioides* spp.**	Th1, Th17, Th2	Tc1	T_EM_	(97–99)
***Histoplasma* spp.**	Th1, Th17	Tc1		(100)
***Paracoccidioides* spp.**	Th1, Th17	Tc1		(101)
***Pneumocystis* spp.**	Th1?	Tc1		(102)
***Talaromyces* spp.**	Th1, Th17			(103, 104)

Different subsets of CD4^+^ T helper cells and CD8^+^ T cells have been shown to participate in immunity against different pathogenic fungi. In some of the pathogenic fungi, different types of memory T-cell development have been documented.

## Pathogen-specific antifungal T cells

### Candida

Anti-*Candida* memory T-cell responses are studied in the context of mucosal infections, vaccine-induced responses, and commensal-specific/pre-exposed T cells in healthy donors. *Candida* is a commensal but opportunistic fungal pathogen that causes disseminated infection under compromised immunity. In a mouse model of oropharyngeal candidiasis (OPC), after resting for 6 weeks following primary infection, the memory CD4^+^ conferred immunity to secondary infection by producing antigen-specific IL-17A responses (91). However, depletion of CD4^+^ T cells did not cause OPC, possibly compensated by developed residual IL-17A expressing memory CD8^+^ T cells and CD3^+^CD4^−^CD8^−^ cells. In a mouse model of *C. albicans* skin infection, IL-17^+^ CD4^+^ T cells were enriched in the skin, which transitioned into sessile CD69^+^/CD103^+^ tissue-resident memory T cells (T_RM_) within 90 days. This suggests that the long-lasting antifungal memory Th17 cells are generated in the non-lymphoid organ, such as the skin (105). Importantly, these T_RM_ cells provided better immunity than migratory Th17 cells following infectious challenges. In a mouse model of vulvovaginal candidiasis, Th17 cells persisted even after the clearance of the yeast but in low numbers by day 30 (106). The vaginal washes showed the presence of IL-17A, IL-23, and β-defensins. Nevertheless, this study did not examine the long-term maintenance of effector/memory CD4^+^ T cells.

The *C. albicans* hypha-specific surface protein antigen, agglutinin-like sequence (Als3)-based NDV-3A vaccine was used for active immunization in a mouse model that prevented *Candida* colonization at vein catheterization site (107), and the mechanisms involve the induction of high levels of anti-rAls3p-N antibodies. Here, the antibody titers persisted 15 days post-boost, interfered with *Candida* colonization at the catheter site, and reduced the fungal burdens in the kidneys. Although the elicitation of CD4^+^ T-cell responses or their persistence was not evaluated in this study, the blocking/inhibitory ability of the antibodies may suggest their potential. In another study, where the NDV-3 vaccine was used in a mouse model of vaccine immunity to vulvovaginal candidiasis, robust antibody responses and immunity were dependent on both T and B cells (108). However, the immunity was assessed 2 weeks following the boost, which may not give a clear understanding of long-lasting memory CD4^+^ T-cell development. Nevertheless, in a human study, intramuscular NDV-3 vaccination of the volunteers induced the durable serum and cervicovaginal antibody titer (anti-Als3) for up to 1 year and provided significant immunity against recurrent vulvovaginal candidiasis (109). This study found significantly higher numbers of Als3-specific cytokine (IFNγ and IL-17A) secreting peripheral blood mononuclear cells (PBMCs) even after day 90 of vaccination. Although this trial evaluated an immunotherapeutic vaccine, whether the vaccination induces the antigen-free (*Candida* reexposure-free) persistence of long-lasting “memory adaptive (T and B) cells” needs to be assessed.

*Candida*-specific memory CD4^+^ T cells in healthy blood donors produced IL-17A and IFNγ, but not IL-10, following restimulation (110). The human *Candida-*specific memory Th17 cells preferentially expressed phenotypic markers, CCR6 and CCR4 (92), which suggests the generation of long-lived anti-*Candida* memory T cells in humans, possibly due to stimulations from commensal microorganisms. Notably, human *Candida*-specific memory CD4^+^ T cells are heterogeneous, produce multiple cytokines, and have unique and shared clonotypes among memory subsets (111). *C. albicans*-specific T_RM_ cells prevented the fungal overgrowth in human skin and oral mucosa by producing IL-17A (105, 112). Similarly, *Candida-*specific IL-9-producing CD4^+^ T cells, Th9, were found enriched in the skin of healthy donors (suggesting their memory phenotype) and have the ability to amplify IFNγ, IL-9, IL-13, and IL-17 by skin-tropic T cells (113). However, gut Th9 cells protect against *Candida* reinfection and mitigate associated pathology (114). Another *Candida-*specific subset of CD4^+^ T cells expressing IL-22 (Th22) is found in humans as memory cells and is increased following infection (115). Further, Th22 seems to provide defense against recurrent vulvovaginitis caused by *Candida* in humans (116), suggesting the formation of mucosal memory Th22 cells. In this line, defective Th22 responses are associated with chronic mucocutaneous candidiasis (117). Although little is known about the fate of pre-existing antifungal memory T cells during co-infection, a recent study suggests the impaired T-cell responses to *Candida* following COVID-19 infection that was associated with diminished inflammatory cytokines release (118).

In the mouse models of mucosal candidiasis, studies have shown that Tc17 cells play a role in both oral and vaginal infections (91, 119). Oral immunization of mice with *C. albicans* under B-cell deficiency induced systemic memory of CD8^+^ T cells and provided protection following the challenge (120). The screening of *Candida*-specific memory CD8^+^ T cells in healthy human blood donors showed a non-classical cytotoxic molecules expression profile, i.e., secretion of granulysin and granzyme K rather than perforin/granzyme B (121). The CD8^+^ T cells were reactive to *C. albicans*, *Candida glabrata*, and *Sporothrix* and expressed lysosomal degranulation markers, CD107a/b, and secreted IFNγ and TNFα, following *ex vivo* stimulation with yeast-loaded dendritic cells.

In one study, the various phylogenetically closer and distant yeast-specific T-cell responses were assessed using PBMCs of humans and found a predominant presence of IFNγ-expressing effector memory CD8^+^ T cells (122). Interestingly, in this study, enriched CD8^+^ T cells were more reactive to filamentous form than the unicellular form of *Candida*. In HIV^+^ patients, the *Candida*-specific activated memory CD8^+^ T cells were accumulated within the oropharyngeal candidiasis (OPC) lesions at the lamina propria–epithelium interface (123). Similarly, *C. albicans*-specific CD8^+^ T cells were found in the blood and nasal mucosa of chronic rhinosinusitis patients, suggesting a possible persistent T cell-mediated mucosal inflammation (124), which may be due to repeated exposure to the antigen.

### Aspergillus

*Aspergillus*, a globally prevalent opportunistic fungal pathogen, causes pulmonary and invasive mycoses following inhalation of spores. Immunity to aspergillosis is primarily associated with the development of memory Th1 cells, while biased Th2 or regulatory responses are linked to exacerbated disease (125–127). Experimental vaccination of mice with conidia, hyphae, crude culture filtrate antigens, or adjuvanted (CpG ODN1862) cell wall glucanase Crf1 protein strongly induced Th1 (IFNγ and IL-2) responses, formed effector or memory T cells, and conferred immunity following the challenge (87, 128–131). In line with the protective role of Th1 responses, IL-4 seems to play a negative role following *A. fumigatus* challenge (127). In chronic rhinosinusitis with nasal polyposis patients, the mycology culture showed the presence of *Aspergillus flavus*, and PBMCs stimulated with aspergillus antigens showed an increased ratio of aspergillus-specific Th17 cells over Tregs, suggesting prior sensitization (132). Further, stimulated PBMC culture supernatant showed elevated levels of IL-17 and IL-10 with reduced TGFβ levels, suggesting the possible associated pathology in these patients. Prior work has shown the negative effect of IL-10 for control of experimental lethal systemic aspergillosis (133), and its overexpression, due to genetic polymorphisms, predisposes to invasive aspergillosis possibly by inhibiting TNFα secretion in hematopoietic stem cell recipients or hematological patients (134, 135). However, IL-10 seems to be protective in regulating exaggerated immune responses and inflammation in allergic bronchopulmonary aspergillosis (136), suggesting that the regulatory role of IL-10 depends on the disease context. In many healthy individuals (10%–30%), multi-*Aspergillus-*specific T cells were found, suggesting their memory potential and feasibility to expand, store, and be used for self-adoptive transfers following hematopoietic stem cell transplantation (137–139). Hematopoietic stem cell transplant patients undergo a period of immunocompromised state, enhancing vulnerability to many opportunistic fungal infections, including aspergillosis. Thus, rapid preventive or therapeutic reconstitution of the functional adaptive immune system is beneficial. Adoptive immunotherapy using either donor-derived (139) or partially HLA-matched antigen-specific T cells can be used to prevent or treat opportunistic fungal infections (140). In a preclinical study, *in vitro* expanded yeast-specific cytokine-producing Th cells have been used to reduce the severity of pulmonary and cerebral forms of aspergillus infections in mice (141).

Although the role of Th17 cells in pulmonary aspergillosis is debated, a recent study suggests the presence of aspergillus-specific Th17 cells that correlated with protective immunity (88), and possible mechanisms may include the formation of inducible bronchus-associated lymphoid tissue (iBALT) structures and development of T_RM_ cells (142). With the use of recombinant aspergillus proteins (Asp f proteins), a study investigated the presence of yeast-specific CD4^+^ and CD8^+^ T cells in healthy non-atopic donors (143). In these individuals, the cytokine production signature suggested the presence of aspergillus-specific memory T cells expressing IFNγ, IL-17A, and to some extent IL-4, possibly due to prior exposure. Importantly, this study showed the presence of *Aspergillus*-reactive IFNγ^+^ T cells for up to 6 months of follow-up observations. However, the persistence of diverse cytokine-expressing T cells as memory in humans needs to be evaluated for their role in immunity or immunopathology following infection.

### Pneumocystis

*Pneumocystis* is another opportunistic fungus that causes infection under immunodeficiency, especially of CD4^+^ T cells and B cells. It is believed that *Pneumocystis* may persist in individuals upon early age exposure without any apparent symptoms, akin to toxoplasma. The reactivation occurs when an individual becomes immunodeficient or severely immunosuppressed, suggesting an active role of memory or effector adaptive immunity to keep the fungus at bay. However, recent studies suggest the possibility of reinfection in immunocompromised individuals (144). Murine models have been valuable in understanding the host–*Pneumocystis* interface for adaptive immunity and recapitulating human primary immune disorders (145). Increasing evidence suggests that B-cell responses are important in the control of *Pneumocystis*. Anti-CD20 mAb therapy in humans enhanced the susceptibility to pneumocystosis, suggesting a critical role of B cells (146). Of note, the CD20 mAb therapy does not deplete mature plasma cells (147), raising questions on mature long-lived plasma cell generation against *Pneumocystis*. It is possible that anti-CD20 mAb therapy leads to functional impairment of antibody-secreting cells. However, in a murine model of pneumocystosis, neither the memory CD4^+^ T cells nor B cells are required for clearance of infection (146). Here, convalescent *Pneumocystis*-specific IgGs were enough to provide immunity. Interestingly, B cells are required for elicitation of antigen-specific CD4^+^ T-cell responses to *Pneumocystis* (148, 149), suggesting a potential cross-talk between these two subsets for immunity against pneumocystosis. Notably, in the simian model of vaccination with *Pneumocystis jirovecii* protease kexin (KEX1), once the B cells are primed, induction of CD4^+^ T-cell deficiency with SHIV infection did not prevent antibody-dependent control of infection, suggesting the persistence of “memory” plasma cells or threshold levels of Ab titers (150). In a murine model, memory CD4^+^ T cells were dispensable for pneumocystosis control, whereas memory CD8^+^ T cells, alveolar macrophages, and *Pneumocystis*-specific IgG contributed to secondary immunity (102). Here, the IgG antibody enhanced the macrophage killing of yeast, while macrophages helped CD8^+^ T-cell recall responses in IFNγ production. IFNγ-stimulated CD8^+^ T cells, in turn, can be potent antifungal cytotoxic cells (13). However, interestingly, CD8^+^ T cells seem to help CD4^+^ T cells with their IFNγ responses. Memory CD4^+^ T cells can indirectly potentiate NK cell functions against infection caused by *Pneumocystis murina*, and depletion of CD4^+^ T cells significantly reduced the accumulation of NK cells and NK-cell mediated immunity (151). In a mouse model of vaccine immunity, immunization with a recombinant fusion protein containing N-terminal 544-aa *Pneumocystis* cross-reactive antigen-1 and trigger factor (TF) induced protective and cross-reactive antibody responses that provided immunity even after memory CD4^+^ T cells were depleted at the time of challenge infection (152). Thus, CD4^+^ T cells seem to have functional duality against *Pneumocystis* infection, first by helping B cells and CD8^+^ T cells to become protective differentiated memory cells and second by secreting proinflammatory cytokines to control the primary infection.

### Cryptococcus

*C. neoformans* is a facultative intracellular opportunistic pathogen commonly associated with AIDS patients due to severe CD4^+^ T-cell deficiency. The CD4^+^ T-cell immunity to cryptococcosis is mainly dependent on Th1 cytokines. Models of vaccination and infection suggested the role of CD4^+^ T cells and their Th1 cytokine profile. *Cryptococcus*-activated CD4^+^ T cells recruited other immune cells, enhanced the phagocytosis, and killed infected cells by CD8^+^ T cells, akin to intracellular bacterial infections (153). Striking effects of Th1-derived cytokines for immunity against cryptococcosis are noticed when genetically engineered *C. neoformans* strain H99 expressing IFNγ (H99-γ) was experimentally used in mice (154). Here, the “vaccinated” mice cleared the infection that was associated with a large influx of leukocytes, enhanced T-cell recruitment, and increased Th1 and decreased Th2-type cytokines following challenge infection. The use of a recombinant strain of *C. neoformans* (H99-γ) as a vaccine strain induced memory CD8^+^ T cells to mediate immunity under CD4^+^ T-cell deficiency, suggesting that antifungal CD8^+^ T cells can compensate CD4^+^ T cells (155). Notably, there was the development of memory T cells and enhanced secondary responses following the challenge. *C. neoformans* chitin deacetylase 2 peptide (Cda2-Pep1) delivered in glucan particle (GP)-based vaccination robustly protected the mice following the challenge, and the immunity was correlated with their MHC-II binding affinity (156). Similarly, multi-epitope vaccine/s may be useful in controlling cryptococcosis (157). In an experimental model, immunization with either cell wall or cytoplasmic protein preparation from *Cryptococcus gattii* induced vaccine immunity after challenge and protection that was associated with enhanced Th1 responses and antigen-specific serum IgG (158). Nevertheless, in such vaccination platforms, the development of memory T cells is not clear. However, *Cryptococcus* antigen-pulsed dendritic cell-based systemic vaccination elicited long-lived memory Th17 cells in the lungs (95). Interestingly, these cells were lung resident T_RM_ cells, produced IL-17A but not IFNγ, and mediated protection against *C. gattii* challenge. Pulmonary infection with *C. neoformans* elicited strong CD8^+^ T-cell responses to control the infection independent of CD4^+^ T cells (15). In this line, immunization with the genetically mutant *Cryptococcus* strain (Δsgl1) that accumulates steryl glucosides led to induction of protective immunity that required either CD8^+^ or CD4^+^ T cells (159). However, these studies did not show memory T-cell development or persistence. In HIV-associated *Cryptococcus* meningitis patients, the clearance of infection was strongly correlated with Th1, not Th2 or Th17, cytokines (IFNγ or TNFα). In contrast, their defective expression led to higher mortality (160), suggesting an importance of type I immunity. Lower frequency of cytokine-producing memory CD8^+^ and CD4^+^ T cells was found in HIV-infected patients with *Cryptococcus* meningitis (CM), but their numbers were increased with more polyfunctional IL-2^+^/IL-17^+^ CD4^+^ T cells and IL-2^+^ CD8^+^ T cells following antiretroviral therapy (ART) in CM-associated immune reconstitution inflammatory syndrome (CM-IRIS) (161) patients, suggesting the pathological role of cryptococcal memory T cells under certain conditions.

### Blastomyces

Mouse models of immunity to blastomycosis suggest that CD4^+^ T cells are essential for controlling primary pulmonary infections. An experimental mouse model of vaccination suggested that Th17 cells expressing IL-17A are the main driver for immunity against pulmonary blastomycosis by activating macrophages and neutrophils (56). A *Blastomyces*-specific fungal antigen, Calnexin, was found to be conserved among multiple fungal pathogens, and vaccination with Adjuplex adjuvant or encapsulated glucan mannan particles seems to induce robust CD4^+^ T-cell responses and immunity (162, 163). Identification of such conserved antigens may help the design of pan-fungal vaccines (164). Although homeostasis of memory CD4^+^ T cells was not studied, the fungal-specific CD4^+^ T cells persisted for 8 weeks and the adoptive transfer of vaccine-induced effector CD4^+^ T cells mediated the immunity following the lethal challenge even after 10 weeks of rest (162), suggesting their potential to become memory. Interestingly, intranasal delivery of vaccine-candidate *Blastomyces* endonuclease-2 (Bl-Eng2) induced T_RM_ cells in the lungs but failed to provide proactive immunity, unlike systemic vaccine-induced migratory CD4^+^ T cells (165). Importantly, Bl-Eng2 is a glycoprotein antigen that has mannose residues that bind to Dectin-2 and a protein backbone with protective CD4 T-cell epitope/peptide (165, 166), thus a vaccine candidate with intrinsic adjuvanticity property. Admixing adjuvants, especially TLR9 (CpG55.2) and Aldeltin (formulation with alum OH) with fungal antigen (Bl-Eng2 peptide) potentiated the vaccine immunity against blastomycosis, which was dependent on type 1 and type 17 cytokine-producing migratory and lung-resident T cells (167). In the mouse model of vaccination under CD4^+^ T-cell deficiency, CD8^+^ T cells could mount sterilizing immunity to lethal pulmonary infection. Immunity was predominantly mediated by IL-17A^+^ CD8^+^ (Tc17) cells (90) with disparate dependency on the type I cytokines (by Tc1 cells), IFNγ, TNFα, and GM-CSF (168); i.e., deficiency of one Tc1 cytokine was compensated by other Tc1 cytokines. Notably, antifungal memory CD8^+^ T cells were long-lasting and persisted stably without plasticity in the absence of vaccine antigen or CD4^+^ T-cell help (16, 169). These memory CD8^+^ T-cell precursors portrayed stem cell-like phenotype and can be fine-tuned by MyD88-Akt-mTOR signaling (78). Additionally, targeting the negative regulator of the TCR signaling molecule, Cblb, could enhance memory CD8^+^ T-cell responses of both Tc1 and Tc17 responses to inactivated vaccine and potentiate immunity following lethal pulmonary challenge (170). Memory Tc17 cells predominantly expressed GM-CSF, and these co-expressing cells potentiated the fungal vaccine immunity without precipitating pathology (171).

### Histoplasma

*Histoplasma* is an opportunistic fungal pathogen and causes disseminated infection in severely immunocompromised patients. Incidentally, most people living in the Ohio-Mississippi river valley endemic regions were reactive to *Histoplasma* (172), suggesting memory T-cell persistence. Memory CD4^+^ T cells contributed to immunity following the secondary infection, and depletion of both CD4^+^ and CD8^+^ T-cell subsets enhanced the infection and decreased survivability (173). The deficiency of IL-10 conferred salutary effects on memory T cell-mediated protection to secondary histoplasmosis (174). The immunity was dependent on T cell-derived TNFα or IFNγ, and the protection conferred by T cells generated under IL-10 deficiency was robust. Although memory responses of IL-17^+^ T cells are not clear, induction of their effector type and immunity has been noticed following vaccination or infection with *Histoplasma* (56, 90, 170, 175). In a model of histoplasmosis, CD8^+^ T cells could compensate for the loss of CD4^+^ T cells for vaccine immunity (176), and the depletion of CD8^+^ T cells compromised the primary immune responses (177). Immunity to histoplasmosis was perforin-dependent and perforin-independent, which included cytokine-mediated (IFNγ or TNα) mechanisms (178). The antigen cross-presentation by dendritic cells seems to be critical for the elicitation of protective antifungal CD8^+^ T-cell responses (14). Although the above studies do not evaluate memory homeostasis, memory responses to histoplasmosis were bolstered by IL-10 neutralization, where fewer CD8^+^ T cells were enough to mediate immunity (174).

### Coccidioides

Coccidioidomycosis or Valley Fever is caused by species of dimorphic fungus, *Coccidioides*, a major cause of mycosis endemic to the southwestern United States. Immunity to Valley Fever is primarily associated with T cells expressing both type 1 and type 17 cytokines (Th1/Th17) (179, 180). The regulatory T cells were associated with persistent coccidioidomycosis in the pediatric population that was recapitulated in resistant vs. susceptible mice (181, 182). Cytokine IL-10 plays a negative role in memory Th1 and Th17 recall responses and immunity following *Coccidioides* infection, but not for the development of memory T cells. Coccidioidomycosis immune donors had polyfunctional T cells composed of both effector and central memory phenotypic cells (183), suggesting their long-term immune role. In a human vaccine study using formaldehyde-killed sperules of *Coccidioides immitis*, no statistical differences in susceptibility to infection were found between placebo and vaccination groups (184). However, the memory T-cell development or their functions between these groups were not clear. Nevertheless, there is an active attempt to improve the vaccine efficacy using a multivalent vaccine against *Coccidioides* infection that required mixed Th1 and Th17 cell-mediated immunity (185, 186). In a mouse model of vaccine immunity using a temperature-sensitive, auxotrophic mutant of *C. immitis*, the adoptive transfers of either CD4^+^ or CD8^+^ T cells from vaccinated mice to recipient mice infected with lethal strain conferred protection, and the immunity was mediated through TNFα (97). In this study, although effector CD8^+^ T cells (2 weeks post-immunization) were used to show protective immunity, the mice were monitored for 50 days following the challenge, suggesting the effector cells’ persistence and possible conversion into memory cells.

### Paracoccidioides

Immunity to paracoccidioidomycosis depends on the CD4^+^ T cells expressing Th1 cytokines, IFNγ, TNFα, and IL-2 (187). The type of T-cell response determines the nature of the disease or the susceptibility, with Th2-dominant responses being non-protective and a mix of Th17/Th22 and Th1/Th2 providing the intermediate protection (188). Experimental vaccination with P10 antigen (of gp43 protein) in Montanide ISA 720, CFA, flagellin, and DODAB adjuvants induced Th1 response and protected against intratracheal challenge infection (189, 190). Here, both effector and central memory phenotypic cells were found to be successfully recalled into the lungs after infection. Similarly, fungal-specific memory CD4^+^ and CD8^+^ T cells were seen in the patients, and low numbers of memory CD4^+^ T cells were associated with relapse of the disease (191). In the model of pulmonary paracoccidioidomycosis, CD8^+^ T cells were induced to control the fungi in the absence of CD4^+^ T cells by secretion of type 1 cytokines (IFNγ and IL-2), and the depletion of CD8^+^ T cells increased the fungal burden with a concomitant increase of non-protective IL-4 and IL-5 cytokines (101). Nitric oxide helps control *Paracoccidioides* infection, and deficiency of NO and CD8^+^ T cells seems to be detrimental to immunity. Interestingly, CD8^+^ T cells enhance the recruitment of TNFα/IFNγ-producing CD4^+^ T cells and the influx of inflammatory cells (192). However, the maintenance of such a response as memory is unclear.

## Cross-reactive antifungal T cells

Cross-reactivity, the recognition of two or more peptides by the same TCR, of T cells has been documented. Cross-reactive antifungal T cells are useful, and identifying the antigens helps in the generation of a pan-fungal T-cell vaccine (193). *C. albicans* existence as a commensal microbe induces memory CD4^+^ T cells. Incidentally, these *C. albicans*-specific cells can cross-react with airborne fungi, like *Aspergillus*, and exacerbate acute inflammatory lung pathologies (194, 195). Similarly, *A. fumigatus* antigen-induced memory Th1 cells were cross-reactive to *C. albicans* (130). T cells specific to *A. fumigatus* were cross-reacted to induce protective immune responses against *Aspergillus* and *Mucorales* sp. infections (138). *A. fumigatus*-specific T cells, in culture, exhibited cross-reactivity with lysates derived from other fungi, including non-fumigatus *Aspergillus*, *C. albicans*, *Penicillium* spp., and *Scedosporium apiospermum* but not with *Aspergillus terreus*, *C. glabrata*, *Fusarium* spp., and *Mucor* spp. (196). Although such distantly related fungi have cross-reactive T cells, high cross-reactivity of T cells was noticed between phylogenetically related *Scedosporium* and *Lomentospora* species, but not with *A. fumigatus* (197). The cross-reactivity by adaptive immunity across kingdoms, i.e., fungus and bacteria, is noticed and exploited for vaccination against *Candida* and *Staphylococcus aureus* (198), including emerging multidrug-resistant *Candida auris* infection (199). Similarly, pan-fungal vaccines can be developed and used against different pathogenic fungi if the shared antigen is identified (163). Although the mechanism of cross-reactivity of fungal T cells is mainly due to shared epitope sequence (163), the broad cross-reactivity of the CD4^+^ T cells may be due to the nature of TCR binding with peptide-MHC class II that allows multiple anchor residues with greater flexibility of amino acid variation (200, 201). The bystander activation *via* TCR-independent mechanisms (202) may also be involved.

## Mechanisms of T cell-mediated fungal control

T cell-mediated control of fungal infections involves mechanisms that are chiefly mediated through the effectual functions of the cytokines they secrete. Additionally, cytotoxic functions, independent of cytokines, of CD8^+^ T have been documented during fungal infections. Here we highlight some of the actions of cytokines/cytotoxic molecules for fungal control.

IL-17A and GM-CSF cytokines at the site of infection protected against fungal pathogens in part by enhancing the neutrophil recruitment to the site of infection (9). Further, these cytokines can activate recruited neutrophils and macrophages to bolster their ability to kill fungal cells. Activated neutrophils and macrophages may exert direct fungicidal activity *via* phagocytosis, degranulation, ROS production, and neutrophil extracellular trap (NET) formation (203). Different granules (primary azurophilic, secondary specific, and tertiary gelatinase) in neutrophils contain different cytolytic molecules (17). The role of the IL-17 axis and neutrophils during dimorphic fungal infections has been reviewed elsewhere (204). IL-17 and IL-22 act on cells to promote STAT3 activation, upregulate Reg proteins, and secrete antimicrobial peptides (AMPs), S100 proteins, and β-defensins from epithelial cells and keratinocytes that destroy the fungal pathogen (205). Further, IL-22 signaling helps in the regeneration of oral epithelial cells and “licenses” IL-17 signaling for resistance against oral mucosal candidiasis (206). AMPs secreted by epithelial cells induce cell wall permeabilization, mitochondrial dysfunction, and osmotic dysregulation in fungi to elicit fungicidal and fungistatic activity (207). Conversely, IL-17A may drive allergic outcomes by enhancing eosinophil recruitment following repeated exposure to *A. fumigatus* conidia (208).

IFNγ, GM-CSF, and TNFα enhance macrophage functions by promoting phagocyte maturation, polarization of macrophages to M1 type, and fungus-killing ability. IFNγ is known to strongly activate phagocytes and their functions against fungi (209). Both IFNγ and TNF induce ROS production from macrophages, which is fungistatic to intracellular fungal pathogens *Histoplasma capsulatum* and *C. immitis* (210, 211). Furthermore, IFNγ promotes rapid acidification of phagolysosomes in macrophages (212), upregulation of MHC-II molecules, and antigen presentation by APCs to elicit T-cell immunity (213). GM-CSF enhances macrophage ROS production and limits intracellular yeast growth by sequestration of zinc (214). GM-CSF deficiency impairs the production of TNFα and IFNγ (215), which are important for the control of intracellular fungal infections. Similarly, TNFα signaling enhances the activation, phagocytosis, and ROS-producing ability of innate immune cells for antifungal functions. Interestingly, early TNFα expression during *C. neoformans* infection increased the fungal burden, reduced mature dendritic cells, and increased Th2 responses (216). TNFα was needed for the maturation and recruitment of DC and the production of IL-12 and IFNγ. However, TNFα prevented biofilm development by *C. albicans* (217), and TNF blockade can enhance opportunistic infections.

Cytotoxic functions of T cells are well documented for CD8^+^ T cells. Antifungal CD8^+^ T cells deploy several antimicrobial granules (mainly granulysin and granzyme K) (121) for direct cytotoxic activity against the fungal pathogen or fungus-infected cells (218, 219). Granzymes can mediate cell death by induction of active caspases, generation of ROS, and mitochondrial damage, while perforin may facilitate their release from the endosomes (220). Although this review focuses on T cells and their memory, antifungal NK cell development is not well documented. NK cells, like CD8^+^ T cells, can produce cytotoxic molecules to kill fungi. The binding of NK cell receptors NKp46 and NCR1 to surface glycans of *Candida* led to degranulation and death of the yeast (221). Similar observations were made with human NK activating receptor NKp30 for the direct killing of *Cryptococcus* and *Candida* (222). Interestingly, “cytotoxic” CD4^+^ T cells can also produce granulysin to mediate the killing of *C. neoformans* (223), and this function was dysregulated in HIV-infected patients.

## CD4^+^ T-cell help for antifungal CD8^+^ T cells

Most fungal infections are caused by opportunistic fungal pathogens under compromised CD4^+^ T cells or their functions. Unlike viral or bacterial models, effector and memory CD8^+^ T-cell homeostasis in fungal infections is poorly understood, and the studies have been done using animal models of fungal vaccine immunity. Attempts have been made to understand the role of antifungal CD8^+^ T cells in the absence of CD4^+^ T cells, a potential avenue to exploit residual immunity for preventive and therapeutic purposes for individuals with CD4^+^ T-cell lymphopenia. Because optimal programming leads to the generation of long-lasting memory CD8^+^ T cells (224, 225) and contributes to fungal vaccine immunity (8), we reason that a potent fungal vaccination can “license” dendritic cells for CD8^+^ T-cell priming independent of CD4^+^ T-cell help. Fungi are decorated, including some secretory, with several potential PAMPs that can bolster the dendritic cell activation and functions (226–228). Therefore, the CD8^+^ T-cell memory imprinting and memory homeostasis following fungal vaccination can be independent of CD4^+^ T-cell help (16, 169). However, evidence from viral and bacterial infections suggests that CD4^+^ T-cell help is essential for eliciting CD8^+^ T-cell responses (229). The mechanisms of CD4^+^ T cells help CD8^+^ T cells involve optimal activation of dendritic cells, enhancement of their phagocytosis, potentiation of antigen processing and presentation, upregulation of costimulatory ligands, and generation of an apt inflammatory *micro milieu* by dendritic cells for naïve CD8^+^ T-cell priming and programming (230, 231). The direct mechanisms of CD4^+^ T cells help involve providing IL-2 for the proliferation of differentiating CD8^+^ T cells and lending co-stimulation through CD40L-CD40 (7). Further, CD4^+^ T cells may help recruit memory CD8^+^ T cells into mucosal surfaces (232). Well-primed CD8^+^ T cells can produce IL-2 (169) and get help in an autocrine manner (233). We also found chemokine receptor-mediated recruitment of effector CD8^+^ T cells into the lungs, which was independent of CD4^+^ T cells following pulmonary challenge in a mouse model of vaccine immunity to pulmonary blastomycosis (90). Recent evidence suggests that the requirement of help from CD4^+^ T cells to CD8^+^ T cells largely depends on their distinct interactions with the dendritic cells during their activation (230, 234). Thus, well-programmed CD8^+^ T cells, endowed with intrinsic memory capacity, can independently bestow their recall responses and immunity (235, 236). Further studies are needed to understand the fungal antigens and the role of dendritic cells in CD8^+^ T-cell priming.

## Memory T cells: An overview

**Figure 1:**
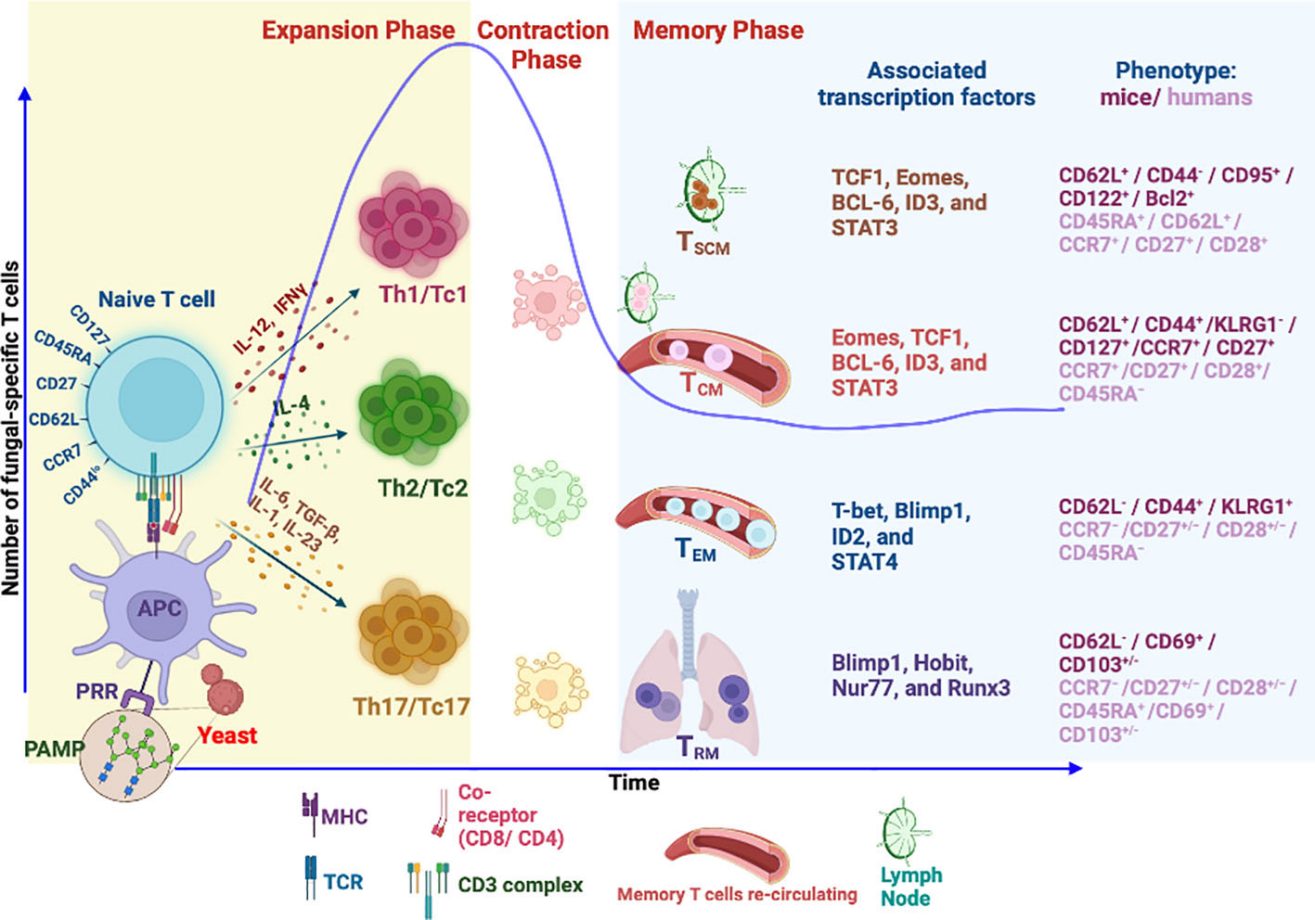
Differentiation and generation of memory T-cell subsets. Following recognition of fungal PAMPs by PRRs, activated antigen-presenting cells (APCs) process the antigen from phagocytosed fungus to load onto MHC molecule. MHC–peptide complex is recognized by cognate TCR of naïve T cells leading to TCR signaling, activation, and differentiation of T cells into different subsets directed by different cytokine milieus. The differentiated T cells accompanied by proliferation during expansion phase secrete inflammatory cytokines to aid in fungal killing. Fungal clearance usually coincides with initiation of T-cell contraction phase where 90% of effector cells die by apoptosis. The remaining cells differentiate to become long-lived memory cells. Memory T cells express unique phenotypic attributes and transcription factors, some of which dictate homing to lymphoid organs (CCR7/CD62L). Tissue-resident memory T cells (T_RM_) continue to reside in tissue of responses. The effector memory T cells (T_EM_) continue to invigilate the pathogen by recirculation between peripheral tissues and blood. Self-renewing central memory (T_CM_) and central memory stem T cells (T_SCM_) are preferentially home to secondary lymphoid organs and serve as “seeders” of secondary effector and memory T cells when needed. PAMPs, pathogen-associated molecular patterns; PRR, pattern recognition receptors; Th, helper T cell; Tc, cytotoxic T cell (CD8^+^ T cell); T_SCM_, stem memory T cells; T_CM_, central memory T cell; T_EM_, effector memory T cell.

The development of immunological memory is the hallmark of vaccination. Following infection or vaccination, the first responders, innate immune cells, exert broadly specific immunity to limit pathogen growth and initiate adaptive T-cell immunity. T-cell recognition is highly antigen-specific and binds to processed antigens/epitopes loaded onto MHC molecules, resulting in their activation, differentiation, and proliferation during the first phase of T-cell response, which is the expansion phase (237). In the ensuing contraction phase, mostly coinciding with the elimination of pathogen or antigen, ~90%–95% of effector T cells [short-lived effector cells (SLECs)] undergo attrition by apoptosis, and the remaining 5%–10% of cells [memory precursor effector cells (MPECs)] differentiate to become long-lived, quickly and robustly responding, memory cells (238) in the memory phase (**Figure 1**). The central paradigm of vaccination is to generate qualitatively superior threshold numbers of memory T cells (237, 239, 240).

Memory T cells are unique and often behave like stem cells in their homeostasis and longevity. Many models of memory T-cell generation are proposed, and one of them suggests that memory programming or imprinting can happen as early as the first antigen encounter (241, 242). Others suggested dynamic and progressive imprinting of memory from a subset of effector cells. In the absence of cognate antigen, memory T cells are quiescent and slow dividing and share many features with naïve T cells (243, 244). However, they are uniquely programmed for stem cell potential and balanced cell apoptosis and proliferation for their steady homeostasis (240, 245), chiefly controlled by cytokines IL-7 and IL-15 (246). Naïve, effector, and memory T cells are differentiated based on their expression of surface and intracellular markers (241, 247–249), which endorse their homing and functional attributes. Memory T cells are a heterogeneous pool of cells derived from multiple clones and fates of polarization and differentiation based on the “one cell multiple fate theory” (250, 251). Hence, these features are imprinted in memory T-cell heterogeneity in homeostatic turnover, effector function, location, and trafficking properties (111, 248, 252).

Classically, memory T cells were divided into two groups, T_EM_ and T_CM_, based on their lymph node homing properties with distinct proliferative renewal and functional properties (240, 241). With the advancement of our understanding and discovery of new markers, memory cells are classified into many groups in both humans and mice. Based on the markers, memory cells are broadly classified as central memory T (T_CM_), effector memory T (T_EM_), tissue-resident memory T (T_RM_), and stem cell memory T (T_SCM_) cells (241, 253–255). Effector memory T cells (T_EM_), CD45RA^−^CD127^+^CD122^+^CD27^−/+^CD62L^−^/CCR7^−^, can migrate into peripheral tissues and exhibit immediate higher effector function with limited proliferation potential during the reinfection. T_CM_ cells, CD45RA^−^CD127^+^CD122^+^CD27^+^CD62L^+^/CCR7^+^, are housed in secondary lymphoid organs with high proliferative potential but weaker effector properties as compared to T_EM_ cells (255, 256). The dogma is that T_CM_ cells are reservoirs or seeders of T_EM_ cells in need. The property of CD62L^lo^/CCR7^lo^ defines the T_EM_ cell exclusion from lymph nodes and migration to peripheral tissues to rapidly exude effector molecules upon antigen encounter. In contrast, T_CM_ cells are CD62L^hi^/CCR7^hi^, allowing them to be preferentially home to lymph nodes (257). Both types of memory cells endow memory homeostasis properties where T_CM_ mediates secondary immune responses for long-term protection and T_EM_ offers instant protection (258). Nevertheless, as alluded earlier, the homeostatic proliferative potential of T_EM_ and T_CM_ cells varies (259).

A newly emerging T-cell memory subset, tissue-resident memory (T_RM_), is restricted to a particular tissue and is identified based on the expression of CD103^+/−^, CD49a, and CD69 (260). The main feature of T_RM_ is their restriction to the tissue (261), possibly due to the expression of CD69 that suppresses the S1P receptor function for lymph node homing (262). Further, TGFβ signaling in these T cells augments the CD103 expression that facilitates latching onto epithelial cells (263, 264). Given the strategic position, T_RM_ cells offer immediate local tissue immunity against invading pathogens (265).

The memory stem T cell (T_SCM_) subset, unique and often considered closest to naïve T cells, exhibits stem cell-like properties with higher self-renewal capacity. Unlike conventional memory T-cell subsets, the T_SCM_ subset maintains a naïve cell phenotype with *multipotency* and serves as a reservoir of memory T cells for a lifetime (266, 267). However, the identity of such unique memory T cells that are fungal-specific is lacking despite the description of antifungal memory cells in humans (111). Further studies are required to reveal the existence of antifungal T_SCM_ in mice and humans.

With our understanding of memory T-cell differentiation and function, the memory cells can be classified in many ways, may depend on the infection or model system, and may be largely due to graded responses during the early programming of effector cells (254, 268–270). Unlike an acute viral infection, in chronic viral infection, the central memory phenotypic T cells poorly develop (271), and high antigen levels induce an exhausted phenotype (272). It should be noted that antifungal memory T-cell homeostasis is poorly defined and not well understood.

## Factors influencing generation of antifungal memory T cells

Our understanding of memory T-cell generation suggests that their attributes are bestowed by early programming during the expansion phase. However, the inflammatory milieu and the antigen persistence can affect their fate. The graded imprinting and epigenetic changes during the effector phase determine the memory T cell fate and the type. Naïve T cells must recognize cognate antigens portrayed on MHC molecules (Signal 1) of dendritic cells. Studies have shown that degree of antigen recognition by naïve or effector cells and antigen persistence (chronic and persistent infections) has a greater impact (273–275). However, this feature can be valuable during vaccine formulations where the antigen is delivered to the site of T-cell activation gradually to enhance the magnitude of their differentiation and expansion. Not surprisingly, booster doses are often given to augment adaptive immune responses, especially with subunit or inactivated vaccines. The costimulatory signal/s (Signal 2) is essential to break the TCR signaling threshold or tolerance to activate the T cells. The role of the classical costimulatory molecule, CD28, in recognizing B7 ligands on antigen-presenting cells is well defined, including during fungal vaccine immunity (276). However, other costimulatory molecules may influence memory T cells’ qualitative and quantitative traits (277), including fungal immunity (278–282). Interestingly, CD28 may play a negative role in Th17 subset differentiation (283), but in the absence of CD28, the differentiation required proinflammatory cytokine signaling (284). Nevertheless, for subunit or less potent vaccine antigen formulations, engaging multiple costimulatory molecules may help potentiate the T-cell responses and eventual memory formation (167, 226). The inflammatory milieu generated by proinflammatory cytokines (Signal 3) is instrumental for effector T-cell differentiation and memory feature imprinting (285). Immunity to different fungal infections needs distinct cytokines produced by T-cell subsets (4), and the deviation from protective T-cell subsets may lead to enhanced pathology and disseminated infections (286–289). Immunity to different fungal pathogens predominantly requires either T cell-derived type I cytokines (IFNγ, GM-CSF, and TNFα) or type 17 (IL-17A/F) responses (80, 290) mainly at systemic and mucosal surfaces, respectively. Nevertheless, following fungal vaccination or infections, both types of subsets are induced at different magnitudes and found to contribute to immunity at variable degrees (8, 22, 168, 291–294). Future studies are necessary to understand the elements of memory T-cell differentiation, homeostasis, and their recall responses for immunity following infection or vaccination.

## Co-stimulatory and coinhibitory molecules influencing antifungal potential memory T cells

Co-stimulatory and coinhibitory receptor molecules present in T cells can fine-tune immune responses to fungal vaccines and infections. While co-stimulation leads to the potentiation of cell signaling, inhibitory signals deliver opposite effects during T-cell activation, thus inhibiting T-cell responses (295). Similarly, coinhibitory receptors present on memory T cells restrict recall responses but preserve memory cells by inhibiting terminal differentiation (296). Interestingly, the expression of coinhibitory molecules on resident memory CD8^+^ T cells (Trm) is an intrinsic property present in their core gene signature (297, 298). Compared to circulatory memory T cells, resident memory T cells expressing high amounts of coinhibitory receptors (2B4, CTLA-4, LAG3, PD-1, and Tim-3) were able to undergo local proliferation following secondary rechallenge (299). Here, we highlight a few studies where the co-stimulatory or inhibitory molecules modulate antifungal T-cell responses.

PD-1 is a coinhibitory molecule expressed in T cells associated with dysfunction, and blocking PD-1 enhances the T-cell functions. Hence, anti-PD-1 mAb administration improved the fungal clearance in a model of persistent cryptococcosis (297). Interestingly, the effect was independent of effector cell numbers and myeloid cell activation, but reduced expression of IL-5 and IL-10 by lung leukocytes and enhanced sustained expression of OX40, a costimulatory molecule, on T cells. Similarly, the blockade of PD-1 and CTLA-4 improves survival during primary and secondary fungal sepsis (300) associated with improved T-cell functions. Signaling lymphocyte activation molecule (SLAM) family members act as co-receptors for T cells fine-tuning immune homeostasis during infections (301). Mutation in SLAM-associated protein (SAP), required for SLAM signaling, results in X-linked lymphoproliferative disease (XLP). A recent study showed that SLAMF1, a member of the SLAM family, was dispensable for T-cell activation and expansion following fungal vaccination, but the fungal immunity was severely compromised (302). This study of vaccine immunity against lethal fungal pneumonia implied that SLAMF1 is mainly important for innate host control of lung fungal overgrowth as well as inflammation, recruitment, or expansion of fungal-specific effector CD4^+^ T cells. Another molecule, CD43, also called sialophorin, is a membrane-bound receptor that exists in two forms with one highly glycosylated on T cells (303, 304). CD43 can be co-stimulatory and inhibitory, and depending on the context, it is known to promote T-cell contraction and reduced memory (305). In the context of cell-to-cell interactions, studies showed that CD43 deficiency led to enhanced homotypic binding of T cells through ligands such as ICAM-1 and fibronectin while augmenting the T-cell proliferation (306, 307). In other studies, pre-activation of CD43 with a mAb reduced the TCR signaling threshold, enhanced the degradation of Cbl, prolonged the TCR signaling, and augmented the T-cell response (308). Our recent study showed the indispensable role of CD43 for Tc17 responses and vaccine immunity to pulmonary fungal infection (309).

T cells typically express one or more co-stimulatory receptors. Thus, understanding the role of their signaling helps design vaccines to bolster qualitatively superior antifungal memory T cells and potentiate their functions for immunotherapeutics. Further, formulations of vaccines for fungal infections should account for the type of T-cell responses desired. Nevertheless, additional studies are warranted to delineate the role of co-stimulatory molecules during fungal infections. Adjuvants in vaccines act to enhance the T-cell stimulatory signals and proinflammatory cytokine production that polarize the T-cell responses (310). Different adjuvants have different characteristics in biasing T-cell response/s. For example, Alum potentiates Th2 responses, while Monophosphoryl lipid A (MPL)/CpG1018 bolsters Th1 responses. For controlling some fungal infections, especially those that are dependent on IL-17 responses, novel adjuvants are necessary.

## Conclusions and future directions

Immunological memory of fungal infections is poorly defined, but their existence and longevity are documented in preclinical and clinical studies. Most studies on T-cell memory came from preclinical model systems of fungal vaccine immunity. It is increasingly evident that T cells play a dominant role in fungal immunity, although other immune elements, including B cells and antibodies, cannot be overlooked. Although the innate cell inflammatory *micro milieu* is key for defining T-cell lineages and their functions for fungal immunity, the direct costimulatory signals or blockade of inhibitory signals delivered to T cells can regulate their effectors and memory cells. Non-canonical T cells such as natural T helper, MAIT, and NKT cell’s role in some fungal infections have been documented, but their persistence as memory cells is not clear (32). Harnessing non-canonical T cells for vaccine immunity can be a new avenue for controlling fungal infections.

The mechanisms of the generation of antifungal memory T cells are not well understood and need in-depth investigations. The studies will be particularly relevant for the development and application of vaccine platforms. As with many different bacterial and viral defense mechanisms, the protective T-cell effectors vary depending on the pathogenic fungi, so the memory T-cell development mechanisms. Further, fungal co-infections and the disease outcomes need thorough studies, such as mucormycosis in COVID-19 patients. Novel findings on the use of fungal PAMPs as adjuvants for vaccines call for an understanding of adjuvanticity properties and their role in the programming of immunological memory. The new concept-based emergence of chimeric antigen receptor T (CAR-T) cells for immunotherapy to treat fungal infections is attractive, and its efficiency or utility needs attention. The plasticity of antifungal T cells has not been clearly understood, and its bases for immunopathology during recall responses and immunity need further evaluation. Further, the identification of phenotypic and functional markers of protective immunological memory T cells would be useful in designing and assessing the potency and efficacy of fungal vaccines.

## Author contributions

JS, SM, and SN conceived, wrote, and edited the manuscript. All authors contributed to the article and approved the submitted version.

## Funding

This study was funded by NIAID-NIH 5R01AI153522 (SN).

## Acknowledgment

Wee used biorender 2022 for genereation of a figure.

## Conflict of interest

The authors declare that the research was conducted in the absence of any commercial or financial relationships that could be construed as a potential conflict of interest.

## Publisher’s note

All claims expressed in this article are solely those of the authors and do not necessarily represent those of their affiliated organizations, or those of the publisher, the editors and the reviewers. Any product that may be evaluated in this article, or claim that may be made by its manufacturer, is not guaranteed or endorsed by the publisher.
